# Investigating the impacts of airborne dust on herbicide performance on *Amaranthus retroflexus*

**DOI:** 10.1038/s41598-024-54134-5

**Published:** 2024-02-15

**Authors:** Firouzeh Sharifi Kalyani, Sirwan Babaei, Yasin Zafarsohrabpour, Iraj Nosratti, Karla Gage, Amir Sadeghpour

**Affiliations:** 1 Department of Plant Production and Genetics, Faculty of Agriculture, University of Kurdistan, Sanandaj, Iran; 2 Crop, Soil, and Environmental Management Program, School of Agricultural Sciences, Southern Illinois University, Carbondale, IL 62901 USA; 3 Department of Plant Production and Genetics, Faculty of Agriculture and Natural Resources, Razi University, Kermanshah, Iran

**Keywords:** Bentazon, 2,2-dioxo-3-propan-2-yl-1H-2λ6,1,3-benzothiadiazin-4-one, Environmental hazards, Herbicide efficacy, Weed, Natural hazards, Agroecology, Plant stress responses, Plant physiology

## Abstract

Dust pollution poses environmental hazards, affecting agriculture through reduced sunlight exposure, photosynthesis, crop yields, and food security. This study explores the interference of dust pollution on herbicide efficacy to control weeds in a semi-arid region. In a factorial experiment conducted in 2019 and replicated in 2020, the interaction of dust and various herbicide applications, including bentazon, sulfosulfuron, tribenuron-methyl, aminopyralid + florasulam, foramsulfuron + iodosulfuron + thiencarbazone, 2,4-D + MCPA, and acetochlor, in controlling *Amaranthus retroflexus* L. were assessed. Dust induced a 9.2% reduction in the total chlorophyll content of *A. retroflexus*, while herbicide application independently led to a 67.5% decrease. Contrary to expectations, herbicides performed better in dust, except bentazon, which caused a 28% drop in plant height and a 29% decrease in total biomass compared to non-dust conditions. Both herbicides and dust exerted suppressive effects on *A. retroflexus's* leaf and stem weights and overall biomass. Despite dust presence, tribenuron-methyl (95.8%), aminopyralid + florasulam (95.7%), sulfosulfuron (96.5%), and foramsulfuron + iodosulfuron + thiencarbazone (97.8%) effectively controlled *A. retroflexus.* These findings indicate that dust's effect on herbicide efficacy is herbicide-dependent but except bentazon, dust generally increased herbicide efficacy and amplified the control of *A. retroflexus*.

## Introduction

Agriculture significantly contributes to the global economy and human health^1,2^. The quantity and quality of agricultural products, including cereal, the primary source of human nutrition, are substantially affected by the unwanted growth of weeds in agricultural fields. Weeds may cause a crop yield reduction from 15% to over 70%^3,4^. On the other hand, the agricultural sector should increase production by at least 70% by 2050 to fulfill the nutritional requirement of the increasing human population^5^. In order to meet the need for increased yields, the importance of weed control is also increasing, with a focus on applying effective herbicides at the correct dosage, at optimal environmental conditions, and at weed growth stages.

Herbicides are essential for controlling weeds in current intensified agricultural systems worldwide^1,6^. The environmental conditions at the time of herbicide application substantially influence the effectiveness of herbicides in controlling weeds. Current studies^7–9^ show that adverse environmental conditions, such as drought and extreme heat, caused by climate change, can indirectly lead to other environmental problems, e.g., the dispersal of airborne dust to different parts of the world. In recent years, the issue of dust has become a central concern in Middle Eastern countries, including Iran and Iraq^10–13^.

Dust storms have become a common environmental issue in many parts of the world, and their impact on agricultural productivity cannot be overstated^14–16^. Airborne dust often occurs in areas prone to soil erosion and an average annual rainfall of less than 100 mm^17–20^. Dust particles are organic and inorganic materials that vary in diameter and size (5.0 ± 0.4 μm). The most important chemical components in dust particles include SiO_2_, CaO, Al_2_O_3_, Fe_2_O_3_, and MgO^21^. Dust storms can carry heavy metals such as Fe, Zn, Cr, Ni, Pb, Cu, Co, and Cd, with the concentration of heavy metals in dust storms in the Middle East during springtime being higher than the exposure thresholds recommended by the World Health Organization^21–24^.

The literature agrees that dust storms can reduce the efficacy of various herbicide active ingredients^17,25^. The adherence of herbicide molecules to dust particles is the primary mechanism by which dust particles reduce the effectiveness of herbicides. The formation of the herbicide-particle complex makes it difficult for the herbicide to penetrate plant tissues, thus reducing its activity^26,27^. In addition to the chemical and physical properties of dust and applied herbicides, the leaf characteristics of the weed influence the particles' inhibitory effect^17,28–30^.

*A. retroflexus* (*Amaranthus retroflexus* L.) has become a persistent problem in corn (*Zea mays* L.) fields in regions where dust storms are expected. Chemical (herbicide) control efforts have often been unsuccessful, possibly due to suboptimal management decisions and the presence of dust particles on this weed's application surface, interfering with herbicides' absorption and effectiveness. While there are few studies that evaluated the interaction of herbicide efficacy with dust^17^ and muddy rain^31^, none focused on real dust conditions in the field, making our research uniquely positioned to address this gap and provide a more accurate understanding of the challenges faced in practical agricultural settings. In addition, no study has yet assessed the herbicides efficacy in controlling *A. retroflexus* plants in fields under the influence of dust particles. Therefore, the objective of the current study was to quantify the interactive effects of airborne dust and various herbicide active ingredients on *A. retroflexus* physiological and morphological traits and control efficacy.

## Material and methods

### Location and experimental procedure

An experiment was conducted in the Agriculture Faculty Research field at the University of Kurdistan in Dehgolan with geographic coordinates of 35° 18′ 51.4296″ N, 47° 18′ 56.3616″ E, and altitude of 1866 m during the 2019 and 2020 growing seasons. The average annual rainfall of this region is 350 mm, and according to the Amberge method, the region's climate is Mediterranean and semi-arid^32^. The physical and chemical properties of the soil at the experimental site are described in Table 1.

**Table 1:** Physical and chemical characteristics of the soil at the experimental site.

Soil texture			Organic matter (g kg^−1^)	Electrical conductivity (ds m^−1^)	Microelements			Absorbable potassium (mg kg^−1^)	Absorbable phosphorus (mg kg^−1^)	pH
Sand (g kg^−1^)	Silt (g kg^−1^)	Clay (g kg^−1^)			Br (mg kg^−1^)	Fe (mg kg^−1^)	Zn (mg kg^−1^)			
142.0	384.0	474.0	7.6	0.5	0.7	2.2	0.8	320.0	12.4	7.6

Before the experiment (in 2018 and 2019), *A. retroflexus* seeds were collected from the fields around the experimental site and stored at room temperature under dry conditions until the experiments started. All the acquisitions were obtained following both national and international protocols, and the plant collection took place under the supervision and authorization of the University of Kurdistan. The authors ensure adherence to all local and national guidelines in this regard. A randomized complete block design experiment with a factorial arrangement and three replicates was applied to assess the interactive effects of herbicides and dust particles on *A. retroflexus* control. The experimental factors were two dust levels (with and without dust) and eight commonly used herbicides (Table 2) in the corn production field. The herbicide selection was based on usage rate and availability for control of broad leaves in corn fields in the dust-affected region. The *A. retroflexus* seeds were planted (100 m^−2^) in 1.5 × 1.5 m plots at a 2 cm soil depth on May 24th, 2019, and May 27th, 2020, five additional plots were designated to ensure consistent and precise dust application. After the emergence of *A. retroflexus* seedlings, all plants other than *A. retroflexus* were eliminated by hand weeding to maintain the redroot plant density of 20 m^−2^.

**Table 2:** Details of herbicides applied in the experiment.

Herbicide active ingredient	Mode of Action	Trade name	Formulation	g ai ha^−1^
tribenuron-methyl (TBM)	ALS^a^ Inhibitor	Granstar^®^	75% DF^d^	22.5
aminopyralid + florasulam (APF)	Auxinic + ALS Inhibitor	Lancelot^®^	450 WG^e^	300 + 150
sulfosulfuron (SSN)	ALS Inhibitor	Apirus^®^	75% WG	36
2,4-D + MCPA (2,4-D)	Auxinic	2,4-D + MCPA	67.5% SL^f^	0.975 + 1300
foramsulfuron + iodosulfuron + thiencarbazone (FIT)	ALS Inhibitor	MaisTer^®^ Power	25% OD^g^	719.2
bentazon (BNT)	PS^b^ II Inhibitor	Basagran^®^	48% SL	960
acetochlor (ACR)	GGPP^c^ Inhibitor	Surpass^®^	76% EC^h^	900

^a^Acetolactate Synthase.

^b^Photosystem II.

^c^Geranylgeranyl Pyrophosphate.

^d^Dry Flowable.

^e^Wettable Granule.

^f^Soluble Liquid.

^g^Oil Dispersion.

^h^Emulsifiable Concentrate.

### Dust preparation

The dust gathered during the spring seasons in western Iran in 2019 and 2020. Following an official metrological announcement, a moistened sponge collected dust from smooth surfaces such as windows and cars after each dust event passed through Syria and Iraq. The collected dust was then mixed to obtain a uniform sample, as reported by Naghib Alsadati et al. (2020). The mixed sample was analyzed for its properties, including particle composition, size, and elemental content (Table 3). The mineralogical and elemental properties of the dust were measured in the chemistry lab. X-ray diffractometry (XRD) technique was applied to measure the dust mineralogical content. The mineral concentrations were standardized and normalized to 100% in the unit^33,34^. It should be noted that this method typically excludes the concentrations of low crystalline and amorphous phases, e.g., organic compounds and volcanic glass.

**Table 3:** Properties of the analyzed dust samples in both experimental years.

Particle size	2019	2020	Elements (ppm)	2019	2020
Sand (%)	1.3	1.2	Cl	90	95
Silt (%)	65.5	64.3	Sr	212	225
Clay (%)	33.2	34.5	Ba	267	274
Mean size (μ)	14.8	15.1	Rb	66	59
Mineralogical compositions (%)			Zr	170	165
Quartz	21.2	20.1	Ni	123	111
Albite	4.9	5.1	V	129	118
Orthoclase	2.2	2.6	Co	31	28
Microcline	1.6	1.2	Cu	38	36
Illite	10	9	Zn	90	95
Kaolinite	3.5	3.7	Ga	12	11
Chlorite	4.6	4.6	Br	5	6
Palygorskite	3.6	3.7	Y	24	23
Calcite	35.2	35.3	Nb	14	14
Dolomite	6.2	6.3			
Gypsum	0.8	1.2			
Halite	0.3	0.3			

Dust was applied on shoots of the *A. retroflexus* plants at the 4–6 leaves stage using a windpump at 3 km hr^−1^ speed. This growth stage was chosen due to the ordinary coincidence of the occurrence of dust at the 4- to 6-leaf stage of *Amaranthus* spp. in the field. Initially, the windpump was calibrated using wheat flour (flour particles are the same size as dust particles). All plants were washed using a water sprinkler before dust application to prevent any other dust interference. Dust was applied at one g m^-2^ at the same size (< 3 microns diameter) and rate measured on the other plants when a dust storm passed through western Iran from Iraq and Syria. A plastic shield was used to prevent dust infiltration into adjacent plots. To ensure consistent and precise dust application, the plants from the additional plots were harvested 15 min after dust application and thoroughly washed with distilled water. The solution from the washed leaves was then taken to the laboratory and subjected to a week-long exposure at 40 °C. After the complete evaporation of water inside the containers, the remaining dust was weighed to confirm the uniform and correct application of dust^35^.

### Herbicide application

Ten minutes after dust application, herbicides were applied in the recommended doses described in Table 2. The herbicides were applied by a rechargeable electric-knapsack sprayer equipped with a flood-jet nozzle (8002 E, Ag Spray Equipment), delivering 250 L ha^−1^ at a pressure of 250 kPa. The spraying speed was 5 km hr^−1^, and the nozzle height was 35 cm above the top of the plant canopy. Check-basin irrigation was applied regularly in each plot based on plant water requirement calculation^36^, avoiding shoots. Plants were visually assessed two weeks after bentazon application and four weeks after other herbicide treatments based on the EWRC rating scale (Table 4)^37^. The last applied treatment was 2,4-D to avoid the adverse effects of tank spry residual on other plots.

**Table 4:** European Weed Research Council (EWRC) rating scale is used to score the level of plant injury following herbicide application.

EWRC score	Crop tolerance	Efficacy (weed kill)	Weed control (%)
1	No effect	Complete kill	100
2	Very slight effects; some stunting and yellowing just visible	Excellent	99.9–98
3	Slight effects; stunting and yellowing; effects reversible	Very good	97.9–95
4	Substantial chlorosis and or stunting; most effects probably reversible	Good–acceptable	94.9–90
5	Strong chlorosis/stunting; thinning of stand	Moderate but not generally acceptable	89.9–82
6	Increasing severity of damage	Fair	81.9–70
7	Increasing severity of damage	Poor	69.9–55
8	Increasing severity of damage	Very poor	54.9–30
9	Total loss of plants and yield	None	29.9–0

### Measurements

To measure the morphological traits of *A. retroflexus*, including leaf weight, stem weight, plant height, and total biomass, five plants from each plot were randomly selected and were catted at the soil surface 21 days after herbicide application. Also, to assess the physiological features of this weed, leaf samples from the five harvested plants were frozen in liquid nitrogen and transferred to the laboratory. A leaf sample weighing 0.1 g was powdered using liquid nitrogen. After adding five mL of acetone 80% and 0.01 g of magnesium oxide, the mixture was centrifuged at a speed of 3000 rpm for 10 min. Then, its absorbance was measured using a spectrophotometer at 663 and 645 nm wavelengths^38^. To determine the soluble protein concentration, 0.2 g of flag leaf was powdered in liquid nitrogen. After adding two mL of Tris buffer (0.1 normal, pH = 4.7, and 10% glycerol), the samples were centrifuged at a speed of 13,500 rpm for 40 min at 4 °C. Then, 990–995 mL of Bradford’s solution was added to 5–10 mL of the produced enzyme solution, and the mixture was placed in a spectrophotometer to measure its absorbance at 595 nm^39^. To determine the proline concentration in leaves, 0.2 g of leaf tissue was powdered with liquid nitrogen. It was then added five mL of sulfosalicylic acid 3%. After centrifuging the mixture for five minutes at 5300 rpm, two mL of the prepared solution, two mL of ninhydrin solution, and two mL of acetic acid were combined, and the mixture was then placed in a hot water bath for one hour at 110 °C. After that, four mL of toluene was added, and its absorbance at 520 nm was measured using a spectrophotometer^40^. To evaluate the concentration of carbohydrates in leaves, 0.1 g of dried and pulverized sample was combined with 10 mL of ethanol 96%, and the mixture was then heated to 100 °C in a hot water bath. The sample was then centrifuged at a speed of 5300 rpm for five minutes, and the supernatant solution was used to calculate the concentration of carbohydrates that are soluble in alcohol, while the residue was used to calculate the concentration of carbohydrates that are soluble in water^41^.

### Statistical analysis

Data were first evaluated for normality of residuals for both years using PROC Univariate in SAS (SAS Institute, 2015) according to the Shapiro–Wilk test so that the residuals were normal, and no transformations were needed. All data were analyzed using a repeated measure approach in PROC Mixed (ANOVA) in SAS (SAS Institute, 2015), in which dust and herbicide factors were considered fixed effects while year and block were considered random effects. Since there were no differences between the data of both years, the mean comparison of the measured traits was carried out on the pooled data of both years. Fisher's least difference (LSD) test was used at *P* ≤ 0.05 to consider the difference between the applied treatments.

## Results and discussion

### Physiological traits

ANOVA was employed to determine the effects of dust and herbicide application on various physiological parameters, such as chlorophyll-a, chlorophyll-b, and total chlorophyll content, protein, proline, and concentrations of water-soluble and alcohol-soluble carbohydrates of *A. retroflexus* (Table 5). The results of the ANOVA revealed that dust alone meaningfully impacted total chlorophyll, protein, proline, and water- and alcohol-soluble carbohydrates; while herbicides alone significantly impacted all physiological parameters. Moreover, dust and herbicide application interaction also affected chlorophyll-b, proline, and water-soluble and alcohol-soluble carbohydrates (Table 5).

**Table 5:** Analysis of variance of dust and herbicides data on physiological traits of *Amaranthus retroflexus*.

Source of variation	Degree of freedom	Mean squares						
		Chlorophyll-a	Chlorophyll-b	Total chlorophyll	Proteins	Proline	Water-soluble carbohydrates	Alcohol-soluble carbohydrates
Block	2	0.31 ns	0.95 ns	1.97 ns	0.45 ns	0.00 ns	9.83 ns	15.62**
Dust	1	3.74 ns	8.70 ns	37.93 *	5.71 **	0.011 **	5182.11 **	29.53 **
Herbicides	7	77.40 **	27.12 **	205.95 **	5.58 **	0.029 **	1943.06 **	59.95 **
Dust × Herbicides	7	1.37 ns	4.47 ns	6.15 ns	0.09 ns	0.007 **	1401.59 **	8.64 *
Error	30	1.46	3.06	5.25	0.48	0.002	32.13	3.85
Coefficient variation	–	10.86	24.49	12.51	24.53	18.77	16.99	12.67

**, *, ns significant at 1 and 5%, and non-significant, respectively.

#### Chlorophyll

##### Chlorophyll-a

**Figure 1:**
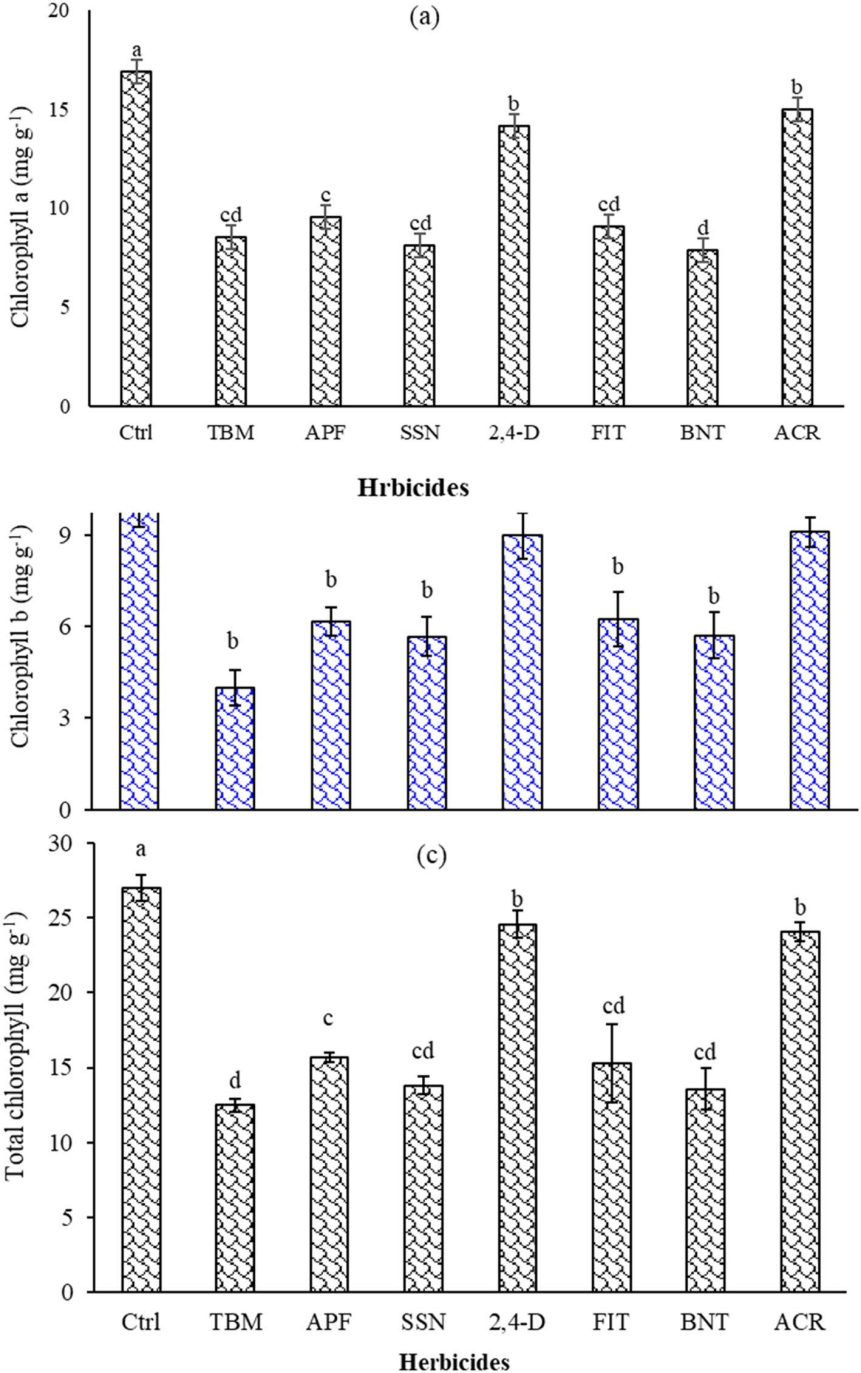
Effect of herbicides on chlorophyll-a (**a**), chlorophyll-b (**b**), and total chlorophyll (**c**) in the *A. retroflexus* leaves. Means with the same letters are not statistically different (LSD = 0.05); bars show the standard error. (Ctrl: untreated control, DC and NDC in figure b: dust and non-dust control, TMB: tribenuron-methyl, APF: aminopyralid + florasulam, SSN: Sulfosulfuron, 2,4-D: 2,4-D + MCPA, FIT: foramsulfuron + iodosulfuron + thiencarbazone, BNT: bentazon, ACR: Acetochlor).

The results indicated that dust did not affect chlorophyll-a content, causing only a 1.47% reduction compared to the no-dust and no-herbicide control (Ctrl). The herbicides bentazon (BNT), sulfosulfuron (SSN), tribenuron-methyl (TBM), aminopyralid + florasulam (APF), foramsulfuron + iodosulfuron + thiencarbazone (FIT), 2,4-D + MCPA (2,4-D), and acetochlor (ACR) reduced the chlorophyll-a content by 53, 51, 49, 43, 46, 16, and 11% respectively, compared to the Ctrl (Fig. 1a). Among the treatments, BNT had the most effect in reducing chlorophyll-a content (Fig. 1a).

##### Chlorophyll-b

Under dust-free conditions, applying TBM, BNT, SSN, and FIT reduced 57, 53, 43, and 32% in chlorophyll-b content compared to the control without dust (NDC) (Fig. 1b). However, herbicides 2,4-D (20 and 1.8% respectively) and ACR (14.6 and 4.9% respectively) did not affect chlorophyll-b content under dusty and non-dusty conditions, compared to the DC and NDC (Fig. 1b). In dusty conditions, TBM, BNT, SSN, FIT, and APF led to reductions of 63, 33, 44, 43, and 59% in chlorophyll-b content, respectively, compared to the DC (Fig. 1b). Additionally, under dusty conditions, chlorophyll-b content decreased with the application of APF (50%) and 2,4-D (18%), while it increased with BNT (29%), compared to when the herbicides were applied under dust-free conditions. Among the treatments, TBM had the most substantial impact (63%) on reducing the chlorophyll-b content of *A. retroflexus* under both dusty and dust-free conditions.

##### Total chlorophyll content (TCC)

Dust reduced the TCC (9%) compared to the non-dust control (NDC; data not shown). Applying TBM, BNT, SSN, FIT, APF, ACR, and 2,4-D reduced TCC by 53, 49, 48, 43, 41, 11, and 9%, respectively, compared to the Ctrl (Fig. 1c). The interaction of dust and herbicide application was not significant.

Previous studies have shown that dust negatively affects plant pigments, such as chlorophyll, due to damage to plant tissue and reduction of pigment concentration^42–47^.

The interactive effect of dust and herbicide on chlorophyll-b may vary by herbicide active ingredient formulation and mechanism of action. A previous study found that dust does not positively or negatively affect TBM efficacy in general^48^. Dust particles can settle on the surface of the leaves and reduce the amount of light that reaches the leaf surface, reducing the herbicide's absorption and translocation. However, this effect is not enough to reduce the overall efficacy of the TBM (Table 6). In addition, TBM is relatively stable and does not break down quickly in the soil or environment^9,49,50^. One of the possible reasons for the lack of effect of dust application may be related to the formulation of TBM and SSN as wettable granules (WG). A wettable granule with 50% active ingredient may contain 42% clay, 2% wetting agent, 2% dispersing agent, 4% inert ingredients, and 50% active herbicide^51^. When these herbicides are applied as dust, the particle size and composition may closely resemble that of the WG formulation. This similarity could potentially enhance the efficacy of the herbicides when they settle on the plant's surface.

Furthermore, the effects of ACR and 2,4-D were associated with increased chlorophyll-b in dusty conditions. The exact mechanism of this observed increase in chlorophyll-b in the presence of dust may be complex and multifactorial. One possible explanation for this unexpected result could be that the dust provided some level of coverage for the target plants and protected them from environmental stresses, such as excessive heat or radiation^52^.

The findings indicated that using BNT led to a considerable reduction in the TCC. The BNT is a photosystem II (PSII) inhibitor (Table 2) that affects the photosynthesis process in plants by disrupting the electron transfer chain in the thylakoid membranes^53^. Dust accumulation on the leaf surface can reduce the amount of light that reaches the leaf, decreasing the photosynthesis rate.

**Table 6:** Visual assessment two week for bentazon and four weeks after herbicide application based on EWRC rating scale.

Herbicide active ingredient	Dust efficacy (%)	Non-dust efficacy (%)
Control	10 g*	0 g
tribenuron-methyl (TBM)	95.8 c	92.1 b
aminopyralid + florasulam (APF)	95.7 c	90.2 c
sulfosulfuron (SSN)	96.5 b	91.8 b
2,4-D + MCPA (2,4-D)	41.2 f	45.4 f
foramsulfuron + iodosulfuron + thiencarbazone (FIT)	97.8 a	96.2 a
bentazon (BNT)	74.5 d	79.7 d
acetochlor (ACR)	53.3 e	58.6 e

*The mean with the same letter is not statistically different (LSD = 0.05).

Moreover, dust accumulation can also reduce the plant's retention and uptake of the herbicide^13,25^. The TCC indicates the plant's photosynthetic activity^54^ and can be affected by the herbicide's mode of action^55^. Since BNT targets photosynthesis, any factor affecting the plant's photosynthetic activity may affect the TCC. Adsorption is a primary mechanism affecting the bioavailability and efficacy of SL herbicides in soil. Herbicide molecules can bind to soil particles, mainly clay, and organic matter, reducing their availability to the targeted plants^56–58^.

#### Soluble protein content (SPC)

**Figure 2:**
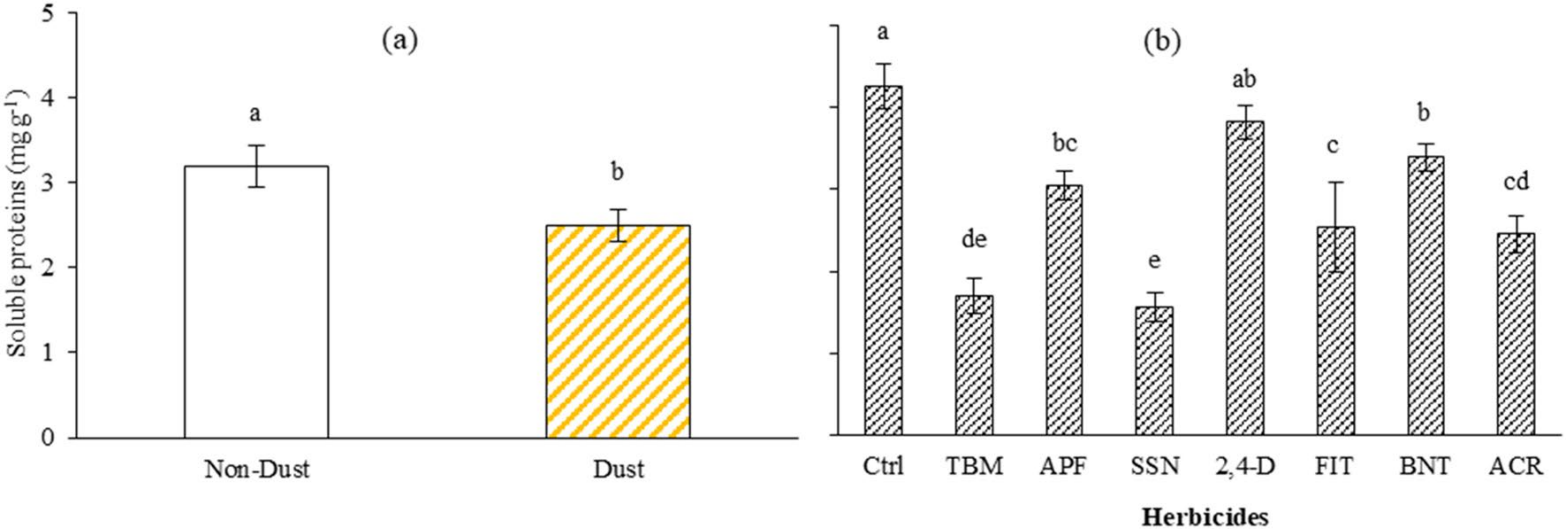
Effect of dust (**a**) and herbicides (**b**) on soluble protein content in the *A. retroflexus* leaves. Means with the same letters are not statistically different (LSD = 0.05), Means with the same letters are not statistically different (LSD), bars show the standard error. (Ctrl: untreated control, TMB: tribenuron-methyl, APF: aminopyralid + florasulam, SSN: Sulfosulfuron, 2,4-D: 2,4-D + MCPA, FIT: foramsulfuron + iodosulfuron + thiencarbazone, BNT: bentazon, ACR: Acetochlor).

Dust decreased the SPC (21%) compared to the NDC (Fig. 2a). The interaction of dust and herbicide did not affect SPC (Table 3). Herbicides, including SSN, TBM, ACR, and FIT, had the highest impact by 63, 60, 42 and 40% decrease compared to the Ctrl, while the effect of BNT had the lowest effect (20% decrease to control) and finally 2,4-D had no result on SPC (Fig. 2b). The APF treatment had an average decrease (28%) of SPC compared to the other herbicides, probably because of the ALS-inhibitor active ingredient florasulam, which comprises one-third of the active ingredient per hectare (g ai ha^-1^) in formulation (Table 2).

The impact on protein synthesis may not be as pronounced with the herbicides. It is hypothesized that the dust-induced stress led to an increase in the production of reactive oxygen molecules such as hydroxyl radicals, free oxygen, hydrogen peroxide, and superoxide, which then interacted with various biomolecules such as proteins, nucleic acids, and lipids, causing damage and mutations to the DNA and breakdown of carbohydrates and proteins^59^. Furthermore, the plant's response to stress may lead to an increase in the activity of protease enzymes, which are responsible for breaking down proteins, and decreased protein production^25^. Also, dust can accumulate on the surface of plant leaves, forming a physical barrier that hinders the exchange of gases and blocks sunlight^60^. Reduced sunlight availability can limit photosynthesis, which is crucial to producing energy and synthesizing organic compounds, including proteins^61^.

Consequently, the plant may experience a decrease in SPC. Furthermore, stomata are tiny openings on the leaf surface that allow the exchange of gases with the atmosphere. When dust particles settle on the stomata, they can clog these openings, impeding the uptake of carbon dioxide (CO_2_) needed for photosynthesis^62,63^. Without an adequate supply of CO_2_, the plant's ability to produce energy and synthesize proteins can be impaired, leading to decreased SPC^64,65^. Among the herbicides, TBM, SSN, and FIT work by inhibiting acetolactate synthase (ALS) (Table 2), which is an enzyme involved in the synthesis of the branched-chain amino acids valine, leucine, and isoleucine^66^. As a result, the plants treated with these herbicides experience a disruption in amino acid production. Since amino acids are the building blocks of proteins, the inhibition of ALS can decrease SPC^67^. On the other hand, herbicides like 2,4-D and BNT have different modes of action that do not directly interfere with amino acid synthesis.

#### Soluble leaf proline content (LPC)

**Figure 3:**
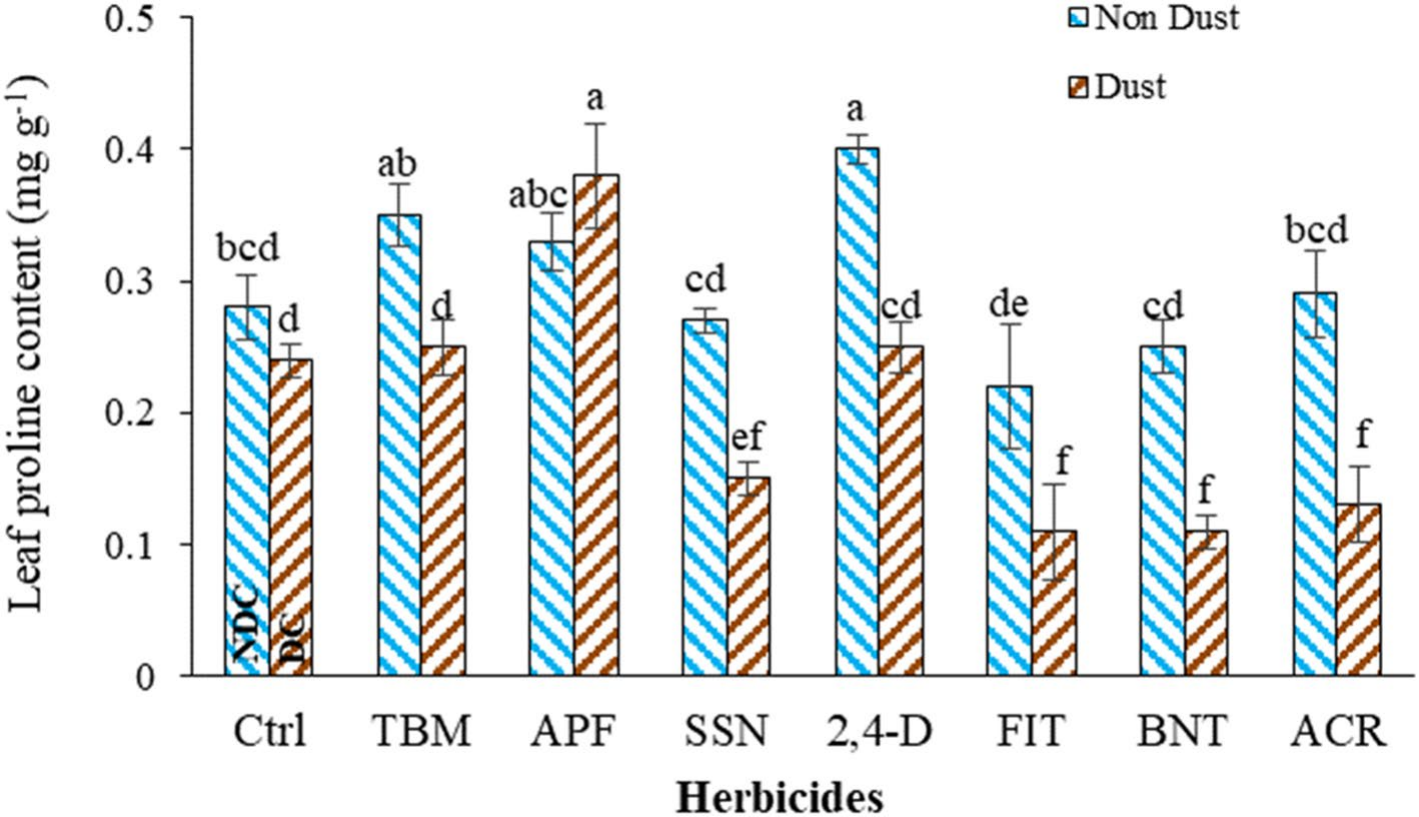
Effect of interaction of dust and herbicides on *A. retroflexus* leaf proline content*.* The mean with the same letter is not statistically different (LSD = 0.05). The bars indicate the standard error. (Ctrl: untreated control, DC and NDC: dust and non-dust control, TMB: tribenuron-methyl, APF: aminopyralid + florasulam, SSN: Sulfosulfuron, 2,4-D: 2,4-D + MCPA, FIT: foramsulfuron + iodosulfuron + thiencarbazone, BNT: bentazon, ACR: Acetochlor).

Dust caused a 14% decrease in the soluble leaf proline content (LPC) compared to the NDC. (Fig. 3). Applying TBM, APF, SSN, 2,4-D, BNT, and ACR changed the LPC compared to the NDC. Herbicides in dusty conditions, including BNT, ACR, FIT, SSN, and 2,4-D, reduced the LPC by 56, 55, 50, 44, and 37% compared to herbicides in non-dust conditions. LPC increased in the presence of 2,4-D, TBM, and APF in non-dust conditions by 42, 17, and 17% compared to the NDC (Fig. 3). All other herbicides exhibited a decrease in LPC in the presence of dust.

Plants accumulate proline as a defensive response to regulate osmotic stress conditions^68–70^ as observed when herbicides are applied in non-dusty conditions. The increase in proline accumulation under herbicide treatment could be attributed to increased protein breakdown. This increased protein breakdown can result in the accumulation of amino acids, including proline, as a byproduct^59^.

#### Soluble carbohydrates in water (SCW) and in alcohol (SCA)

**Figure 4:**
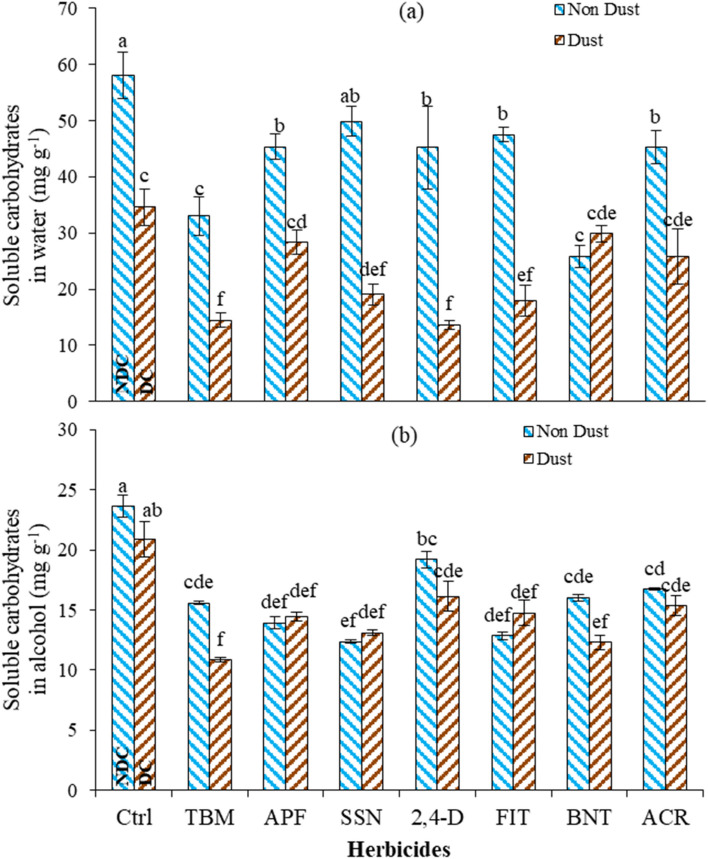
Effect of interaction of dust and herbicides on soluble carbohydrates content in water (**a**) and alcohol (**b**) of *A. retroflexus* leaves*.* The mean with the same letter is not statistically different (LSD = 0.05). The bars indicate the standard error. (Ctrl: untreated control, DC and NDC: dust and non-dust control, TMB: tribenuron-methyl, APF: aminopyralid + florasulam, SSN: Sulfosulfuron, 2,4-D: 2,4-D + MCPA, FIT: foramsulfuron + iodosulfuron + thiencarbazone, BNT: bentazon, ACR: Acetochlor).

Herbicides in the presence of the dust, including 2,4-D, FIT, SSN, TBM, APF, and ACR, resulted in a reduction in the SCW by 69, 62, 61, 56, 37, and 43%, respectively, compared to the herbicide application in non-dust condition and 76, 69, 67, 75, 51, and 55% compared to NDC (Fig. 4a). Herbicides' effect in dusty conditions was increased (Table 7), and the amount of SCW was severely reduced except for BNT, APF, and ACR, which was not statistically different compared to DC. In dusty conditions, TBM, 2,4-D, SSN, and FIT had the lowest amount of SCW. The herbicides and dust particles and their interaction affected the SCA, and the result was partially similar to the SCW obtained with less sensitivity (Fig. 4b).

**Table 7:** Variance analysis of the effect of dust and herbicides on morphological traits in *Amaranthus retroflexus.*

Source of Variation	Degree of freedom	Mean squares			
		Leaf weight	Stem weight	Total biomass	Height
Block	2	0.84 **	0.02 ns	0.001 ns	3.14**
Dust	1	0.43 **	1.07 **	0.29 **	0.002 ns
Herbicides	7	0.74 **	4.95 **	9.53 **	123.07 **
Dust × Herbicides	7	0.13 *	0.16 **	0.16 **	2.15 **
Error	30	0.05	0.04	0.01	0.56
Coefficient variation	–	15.55	10.81	8.90	9.09

**, *, ns significant at 1 and 5%, and non-significant, respectively.

The reduction of soluble carbohydrates in the presence of dust and herbicides could be attributed to several factors. Dust can block sunlight from reaching the plant's leaves. Sunlight is crucial for photosynthesis, providing the energy needed^71^. If the dust layer is thick enough, it can reduce the light reaching the leaves, decreasing photosynthesis and resulting in fewer soluble carbohydrates. Dust particles may contain pollutants, heavy metals, or other harmful substances that can negatively impact plant metabolism. When plants are under stress, their metabolic processes, including carbohydrate synthesis, can be disrupted, decreasing soluble carbohydrates^72,73^.

The enzymes responsible for the regeneration and synthesis of carbohydrates and the Calvin cycle, such as ribulose diphosphate carboxylase, fructose diphosphate phosphatase, NADP glyceraldehyde 3-phosphodihydrogenase, phosphoribulokinase, and pseudoheptulose diphosphate phosphatase, are activated by appropriate light intensity, and the reduction in light due to the dust and shade can negatively impact their function and ultimately reduce the concentration of soluble carbohydrates in water^74^.

Meanwhile, dust accumulation on the leaf surface can reduce the amount of light reaching the chloroplasts, where photosynthesis occurs. Consequently, photosynthetic activity can be impaired, leading to decreased production of carbohydrates, including soluble carbohydrates. The herbicides may exacerbate this effect by further compromising photosynthetic processes. This limitation in carbon dioxide availability can decrease the production of soluble carbohydrates. Also, each herbicide has a specific mode of action that affects plant physiology differently. It is possible that the herbicides themselves directly or indirectly influence carbohydrate metabolism, leading to reduced soluble carbohydrate concentrations in water^60,61^.

Overall, the combination of dust accumulation, physical stress, altered herbicide efficacy, impaired photosynthesis, and potential herbicide interactions with dust particles likely contribute to the observed reductions in soluble carbohydrates in water in the presence of dust conditions. However, it is essential to note that specific interactions between dust, herbicides, and the physiology of *A. retroflexus* should be investigated further to gain a more comprehensive understanding of these effects.

### Morphological traits

#### Leaf dry weight (LDW)

**Figure 5:**
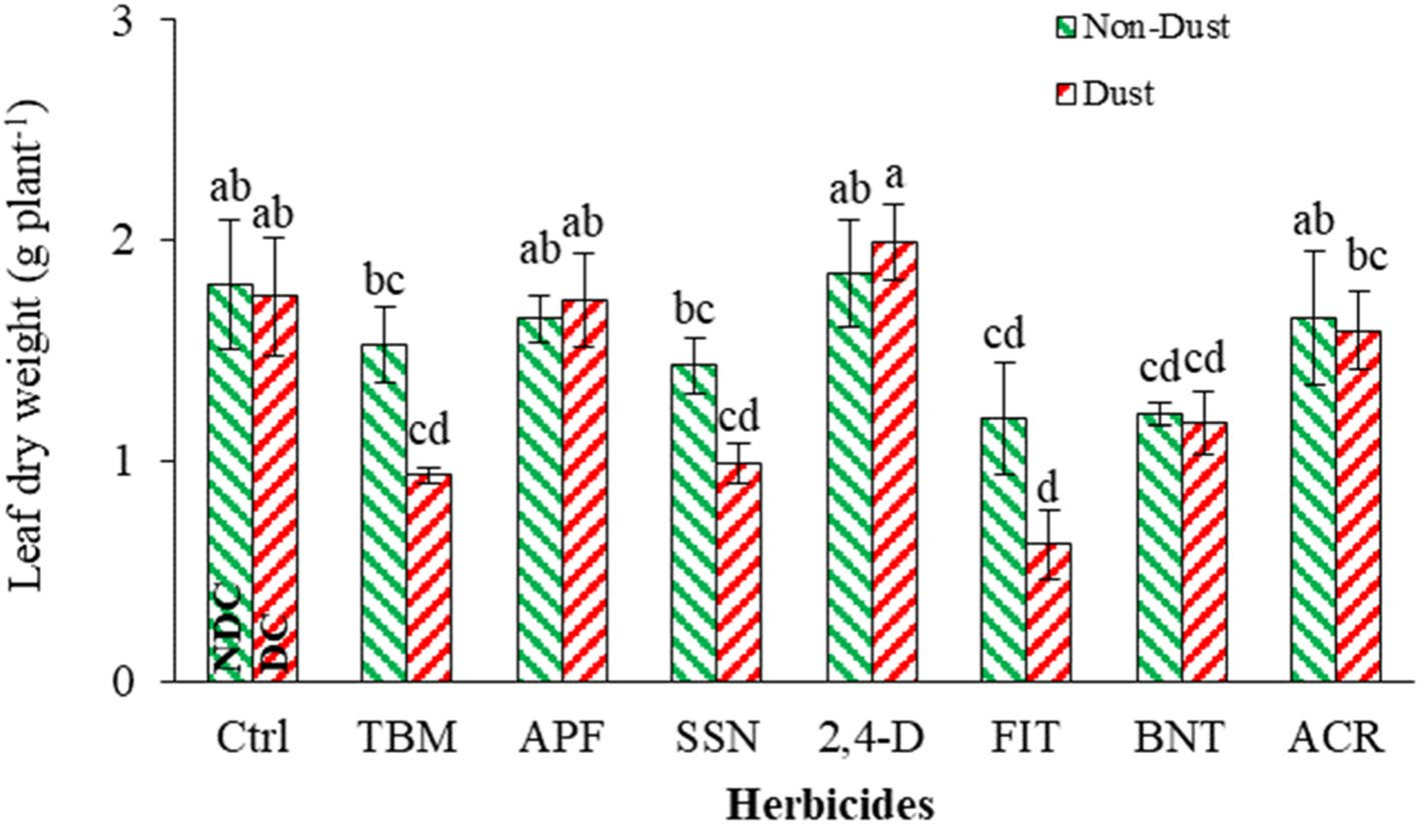
Effect of dust and herbicides interaction on *A. retroflexus*'s leaf dry weight. The mean with the same letter is not statistically different (LSD = 0.05). The bars indicate the standard error. (Ctrl: untreated control, DC and NDC: dust and non-dust control, TMB: tribenuron-methyl, APF: aminopyralid + florasulam, SSN: Sulfosulfuron, 2,4-D: 2,4-D + MCPA, FIT: foramsulfuron + iodosulfuron + thiencarbazone, BNT: bentazon, ACR: Acetochlor).

The result showed that dust, herbicides, and their interactions affected leaf dry weight (LDW), stem dry weight (SDW), and total biomass of *A. retroflexus*—also, herbicides alone and the herbicide in the presence of the dust interaction affected plant height (Table 5). Herbicides TBM, SSN, FIT, and BNT in the presence of the dust reduced LDW by 46, 43, 64, and 32%, while 2,4-D, ACR, and APF had no meaningful effect on LDW compared to DC (Fig. 5). Among the applied treatments, 2,4-D did not affect LDW compared to both controls (NDC and DC), while FIT, TBM, SSN, and BNT had the most effect in the presence of dust by reducing 65, 48, 45, and 35% of LDW compared to NDC (Fig. 5).

Plants can compensate for specific environmental stresses by adjusting their growth patterns^75,76^. *A. retroflexus* may have altered its resource allocation in the presence of dust, shifting resources toward maintaining LDW while reducing other growth parameters, as observed in SDW reduction. This compensatory response could help the plant maintain its structural integrity and support survival^77,78^. Also, dust accumulation on the leaf surface can disrupt the distribution of resources within the plant. The resources redistributed by *A. retroflexus* may prioritize allocation towards LDW while compromising other growth parameters. This redistribution may have allowed the plant to maintain its overall biomass despite the adverse effects of dust on other growth processes^79^.

Each herbicide has a specific mode of action, which determines how it affects plant growth and development. The herbicides FIT, TBM, and SSN may have more potent^80,81^ or targeted modes of action (Table 2) that direct impact processes associated with LDW, such as cell division, elongation, or biomass accumulation^82,83^. On the other hand, the mode of action of 2,4-D may be less effective, or hormesis may be affecting LDW, resulting in a minor impact^84–86^. Also, different herbicides can have varying levels of efficacy on different plant species. *A. retroflexus* may be more susceptible to the FIT, TBM, and SSN herbicides than 2,4-D^85^. The specific biochemical pathways these herbicides target may be more critical for LDW accumulation in *A. retroflexus*.

#### Stem dry weight (SDW)

**Figure 6:**
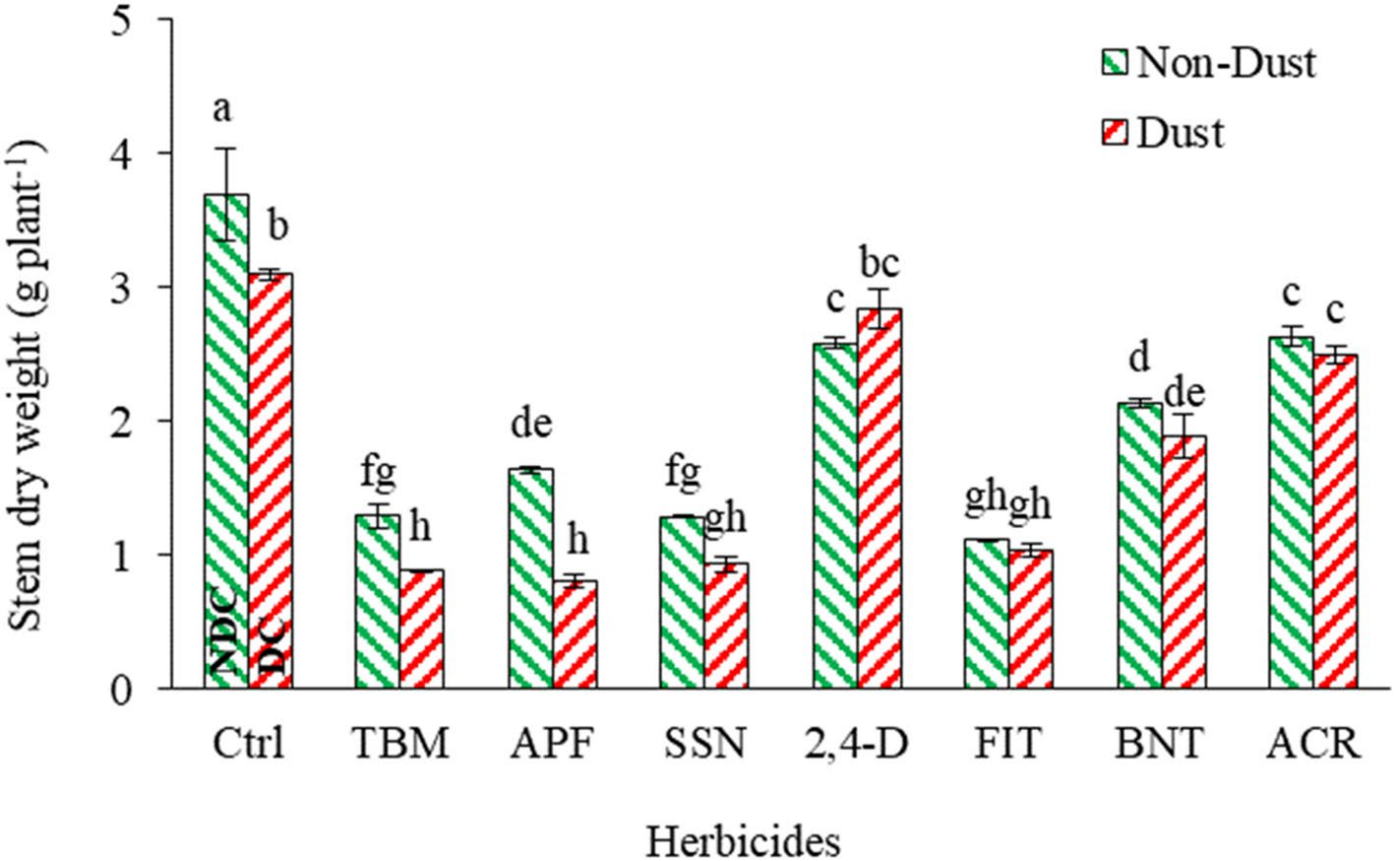
Effect of dust and herbicides interaction on *A. retroflexus*'s stem dry weight. The mean with the same letter is not statistically different (LSD = 0.05). The bars indicate the standard error. (Ctrl: untreated control, DC and NDC: dust and non-dust control, TMB: tribenuron-methyl, APF: aminopyralid + florasulam, SSN: Sulfosulfuron, 2,4-D: 2,4-D + MCPA, FIT: foramsulfuron + iodosulfuron + thiencarbazone, BNT: bentazon, ACR: Acetochlor).

The addition of dust decreased SDW by 16% compared to the NDC for the untreated control plants. The application of herbicides under non-dusty conditions, such as FIT, TBM, SSN, APF, BNT, 2,4-D, and ACR, resulted in reductions in SDW by 69, 65, 65, 55, 42, 30, and 28%, respectively, compared to the NDC. Furthermore, when TBM, APF, SSN, FIT, BNT, and ACR were applied in the presence of dust, there was a decrease in SDW by 71, 73, 69, 66, 39, and 19%, respectively, compared to the control in dusty conditions (Fig. 6). This result indicates that even in the presence of dust, TBM, APF, SSN, and FIT maintained their effectiveness in suppressing *A. retroflexus*'s growth and biomass accumulation, albeit with some variations in the extent of reduction compared to the dust-free conditions.

Research has indicated that dust reduces the stems and branches and various plant parts' fresh and dry weight, which could be attributed to decreased chlorophyll content and photosynthetic processes^87,88^.

The impact of dust on reducing the SDW is more significant than its impact on the root^89^, possibly because the plant's natural photosynthetic activities can meet the root's needs before the dust stress^89^. However, dust may slow the photosynthetic process, and as a result, the products produced by photosynthesis tend to move more toward the leaves and roots^89^.

The variations in the percentage reductions of SDW among different herbicides can be attributed to several factors, including their specific modes of action, effectiveness on *A. retroflexus*, interactions with dust particles, and potential differences in plant sensitivity to these herbicides. Notably, the percentage reductions in SDW reflect the overall impact on plant growth and biomass accumulation. The observed reductions indicate that the tested herbicides have the potential to control the growth of *A. retroflexus*, both in non-dust and dusty conditions, although the presence of dust may influence the effectiveness.

#### Plant height and total biomass

**Figure 7:**
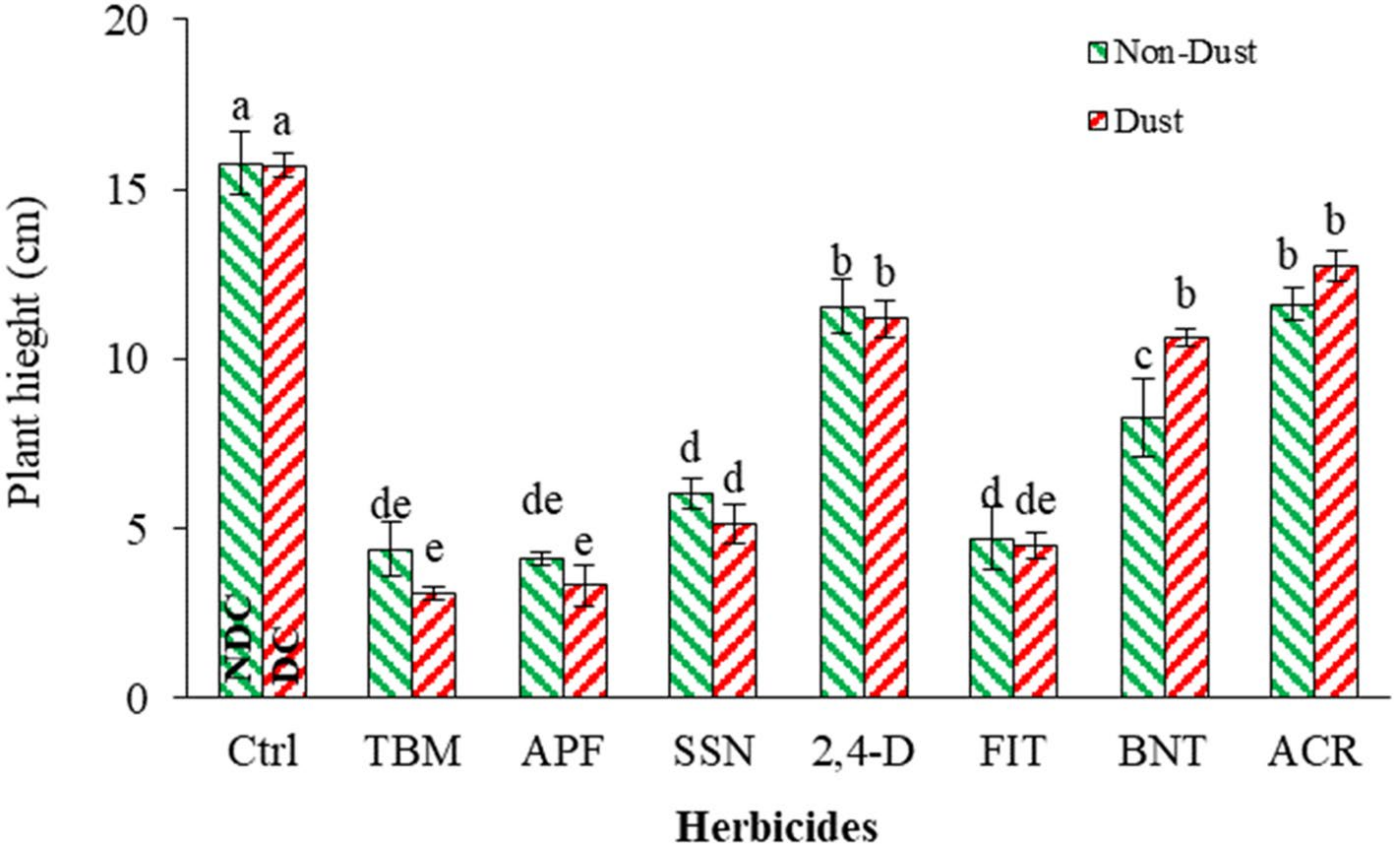
Effect of dust and herbicides interaction on the *A. retroflexus* height. The mean with the same letter is not statistically different (LSD = 0.05). The bars indicate the standard error. (Ctrl: untreated control, DC and NDC: dust and non-dust control, TMB: tribenuron-methyl, APF: aminopyralid + florasulam, SSN: Sulfosulfuron, 2,4-D: 2,4-D + MCPA, FIT: foramsulfuron + iodosulfuron + thiencarbazone, BNT: bentazon, ACR: Acetochlor).

The plant height of *A. retroflexus* was similar in the presence of dust and under dust-free conditions (Table 5). In dust-free conditions, APF, TBM, FIT, and SSN (were not different), BNT and 2,4-D (were in the same group), and ACR (had the slightest effect) caused reductions in plant height of 74, 72, 70, 61, 47, 26, and 26%, respectively, compared to the NDC. Also, in the presence of dust, these herbicides reduced plant height, with significant of 78, 80, 71, 67, 32, 28, and 18%, respectively, compared to the DC (Fig. 7). Among the applied treatments, TBM and APF in the presence of dust had more effect, and plant height decreased (8%) compared to when applied in no dusty condition. While FIT, ACR, SSN, 2,4-D, and APF effects did not change in plant height reduction. Furthermore, BNT application in dusty conditions had a lower effect on plant height decline (Fig. 7).

**Figure 8:**
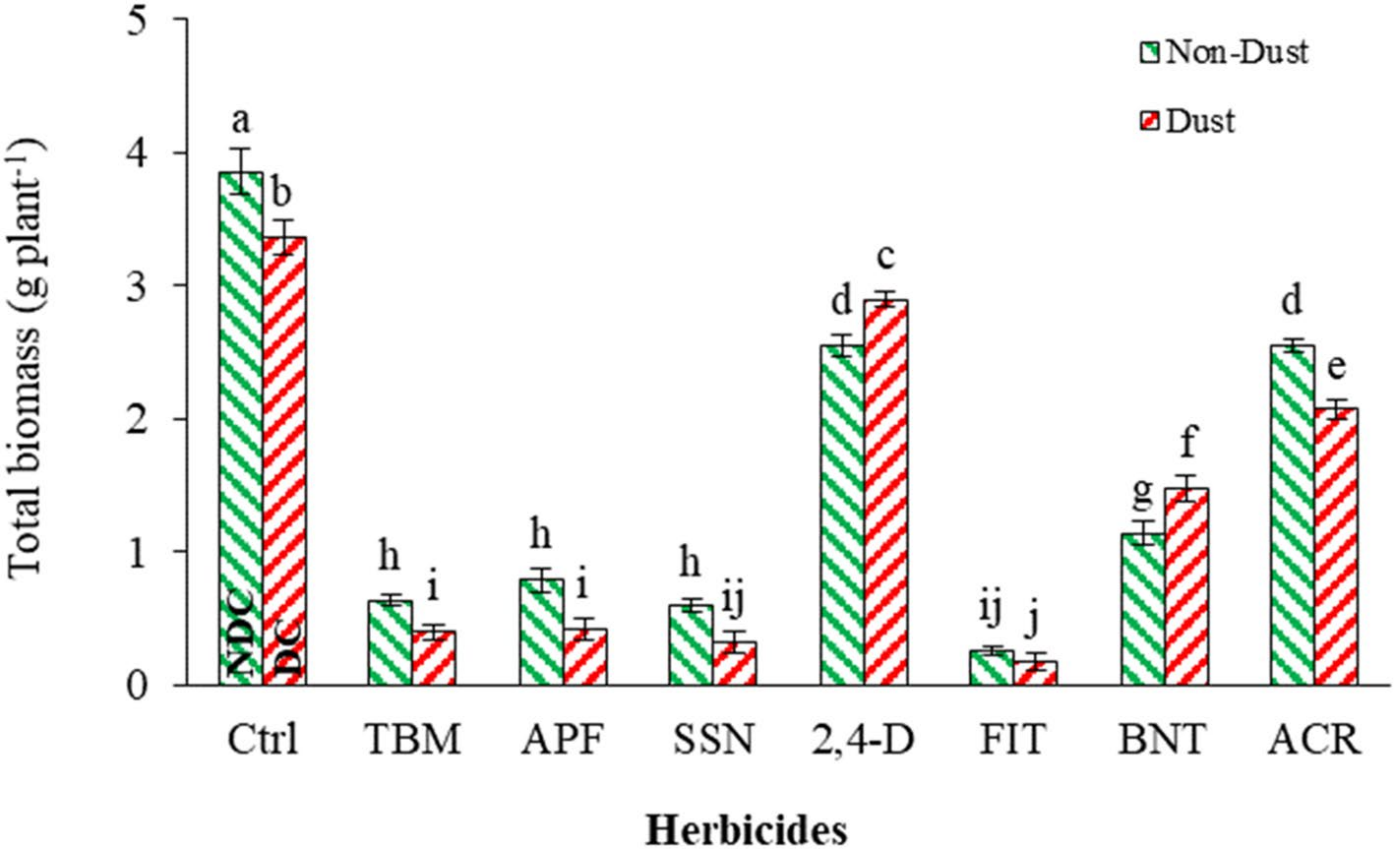
Effect of interaction of dust and herbicides on *A. retroflexus* total biomass. The mean with the same letter is not statistically different (LSD = 0.05). The bars indicate the standard error. (Ctrl: untreated control, DC and NDC: dust and non-dust control, TMB: tribenuron-methyl, APF: aminopyralid + florasulam, SSN: Sulfosulfuron, 2,4-D: 2,4-D + MCPA, FIT: foramsulfuron + iodosulfuron + thiencarbazone, BNT: bentazon, ACR: Acetochlor).

Adding dust reduced the total biomass of *A. retroflexus* by 12%, DC, compared to the NDC. Also, in dust-free conditions, FIT, SSN, TBM, APF, BNT, ACR, and 2,4-D decreased total biomass by 93, 84, 83, 79, 70, 34, and 34%, respectively, compared with NDC. However, these herbicides in the presence of the dust had a different reaction and decreased total biomass by 94, 90, 88, 87, 56, 38, and 13%, respectively, compared with dusty condition control (Fig. 8).

In the presence of dust, TBM, APF, SSN, and ACR decreased biomass compared to NDC, attributed to previously discussed factors, including:

- Increased herbicide retention: Dust particles on the leaf surface can act as physical barriers, potentially enhancing the retention and adherence of herbicide droplets or residues. This increased retention of herbicides in the presence of dust could lead to higher concentrations and prolonged exposure of *A. retroflexus* to the herbicides, resulting in more suppression of its growth and biomass accumulation.
- Enhanced herbicide efficacy: Dust particles may interact with herbicides and alter their properties, potentially enhancing their effectiveness. These interactions can affect herbicide distribution, uptake, translocation, or metabolism within the plant. The combined effects of dust and herbicides such as TBM, APF, SSN, and ACR may result in synergistic or additive effects, leading to a more pronounced reduction in *A. retroflexus* biomass than in non-dusty conditions.
- Stress amplification: Dust particles can induce plant stress by causing physical damage, blocking light penetration, and disrupting stomatal function. Combining stress factors and herbicide action can amplify *A. retroflexus*'s response to dust-induced stres*s*. The cumulative effects of stress from dust particles and herbicides may result in a greater reduction in total biomass than herbicide application in non-dusty conditions.
- Interference with physiological processes: Dust accumulation can interfere with critical physiological processes in plants, such as photosynthesis, water uptake, and nutrient absorption. The presence of dust may exacerbate the impact of herbicides on these processes, further compromising the growth and biomass accumulation of *A. retroflexus*. This interference with physiological processes can contribute to more decline in total biomass under dusty conditions.

As observed in the results, 2,4-D and BNT in the presence of the dust lost their impact on the total biomass of *A. retroflexus* by 13 and 29% compared with non-dusty condition herbicide application, which could be attributed to several factors, including:

- Reduced herbicide deposition: Dust particles on the leaf surface can interfere with the deposition of herbicide droplets or residues. Dust particles may create a physical barrier that hinders contact between the herbicide and the target weed. Consequently, some herbicides may not effectively reach the intended target, leading to reduced efficacy.
- Impaired herbicide absorption: Dust particles can impact the absorption of herbicides by the weed. When dust is present, it may interfere with the penetration of herbicide molecules through the leaf cuticle or hinder their movement within the plant tissues. This interference can reduce herbicide absorption by *A. retroflexus*, decreasing the effectiveness of 2,4-D and BNT in controlling weed growth.
- Dust-induced physiological stress: Dust accumulation on the leaf surface can induce physiological stress on plants. This stress can disrupt various plant processes, including photosynthesis, water balance, and nutrient uptake. Such physiological stress can weaken *A. retroflexus*'s overall vigor and health, making it less responsive to herbicide treatment. The combination of dust-induced stress and herbicide application may reduce the efficacy of 2,4-D and BNT on *A. retroflexus*.
- Dust-mediated herbicide degradation: Dust particles may contain compounds or microbes that could interact with herbicides and potentially degrade their active ingredients. These interactions may alter the chemical properties or stability of 2,4-D and BNT, decreasing their effectiveness against *A. retroflexus*.
- Differential susceptibility of *A. retroflexus*: It is also possible that *A. retroflexus* exhibits a lower sensitivity or resistance to 2,4-D and BNT under dusty conditions than in non-dusty conditions. Dust-induced stress or other dust-related factors may confer tolerance or reduced susceptibility to these herbicides in *A. retroflexus*, reducing their efficacy.

It is important to note that the specific interactions between dust, herbicides, and *A. retroflexus* can be complex and influenced by various factors, including the composition of the dust, formulation of the herbicides, plant physiology, and environmental conditions. Further research and experimentation would be required to gain a more comprehensive understanding of the underlying mechanisms responsible for the observed reduction in the efficacy of 2,4-D and BNT in the presence of dust.

## Conclusion

In conclusion, dust significantly impacted *A. retroflexus*'s physiological and morphological traits. While previous research has recognized the negative impact of dust on plant physiology, affecting processes like photosynthesis, nutrient uptake, and growth parameters, which subsequently diminish herbicide efficacy, our recent study has revealed an interesting finding. Despite its harmful effects, dust may also have the unexpected benefit of enhancing the efficacy of certain herbicides. Dust particles reduced chlorophyll content, potentially affecting herbicide efficacy. Herbicides with different modes of action exhibited varied responses in the presence of dust. Dust accumulation led to a decrease in soluble protein content and soluble carbohydrates, likely due to reduced photosynthesis and impaired gas exchange. The plant's compensatory response focused on maintaining leaf and stem dry weight while reducing other growth parameters. The combination of dust and herbicides reduced total biomass, with potential factors including increased herbicide retention, enhanced herbicide efficacy, stress amplification, and interference with physiological processes. However, the effectiveness of 2,4-D and BNT on total biomass was compromised in the presence of dust, potentially due to reduced herbicide deposition, impaired absorption, dust-induced stress, dust-mediated degradation, or the differential susceptibility of *A. retroflexus*. Farmers in areas prone to dust accumulation should adopt regular dust management techniques, such as pre-herbicide, rain irrigation, and the utilization of adjuvants, to mitigate the efficacy of herbicides against weeds. Researchers and policymakers must also collaborate in developing effective weed management strategies in such environments.

## Data Availability

The data that support this study will be shared upon reasonable request to the corresponding author.

## Abbreviations

TMB: Tribenuron-methyl
BNT: Bentazon
FIT: Foramsulfuron + iodosulfuron + thiencarbazone
ACR: Acetochlor
SSN: Sulfosulfuron
APF: Aminopyralid + florasulam
TCC: Total chlorophyll content
SPC: Soluble protein content
LPC: Soluble leaf proline content
SCW: Soluble carbohydrates in water
SCA: Soluble carbohydrates in alcohol
LDW: Leaf dry weight
SDW: Stem dry weight
